# Embryo viability and hatchability under eggshell-free culture in White Leghorn and Barred Plymouth Rock chickens

**DOI:** 10.1016/j.psj.2026.107467

**Published:** 2026-07-17

**Authors:** Kie Murai, Hiroshi Kagami

**Affiliations:** Faculty of Agriculture (Ina Campus), Shinshu University, 8304 Minamiminowa-Village, Kamiina-County, Nagano, 399-4598, Japan

**Keywords:** Eggshell-free culture, Chicken embryo, Embryo viability, Hatchability, Genetic background

## Abstract

Eggshell-free (*ex ovo*) culture systems enable direct observation and manipulation of avian embryos, but embryo viability and hatchability under these conditions remain variable. This Research Note evaluated embryo viability during culture and hatchability of White Leghorn (**WL**) and Barred Plymouth Rock (**BPR**) chicken embryos under eggshell-free culture. A total of 28 embryos from two independent experiments were analyzed (WL, *n* = 13; BPR, *n* = 15). Embryo viability during culture was analyzed using the Kaplan-Meier method, with embryo death or developmental arrest treated as an event and hatching treated as a censored observation at the day of hatching. Embryo viability during culture did not differ significantly between WL and BPR embryos by the log-rank test (*p* = 0.472). Hatching was observed in 3 of 15 BPR embryos (20.0%; 95% CI, 4.3-48.1%) but in none of the 13 WL embryos (0.0%; 95% CI, 0.0-24.7%); however, hatchability did not differ significantly between breeds by Fisher’s exact test (*p* = 0.226). All 3 hatched chicks died from undetermined causes within 2 wk. after hatching; these post-hatching observations were treated descriptively and were not included as primary endpoints. These results support the separate assessment of embryo viability during culture and hatchability under eggshell-free culture conditions. Although statistically significant breed differences were not detected in the present dataset, the observation that hatching occurred only in BPR embryos highlights the need to consider breed, genetic background, or source-flock-related factors as potential contributors in future evaluations of eggshell-free culture systems.

## Introduction

Eggshell-free (*ex ovo*) culture systems provide a valuable platform for studying avian embryonic development by enabling direct observation and manipulation (Perry, 1988; Tahara and Obara, 2014).

However, embryonic development under eggshell-free conditions remains unstable, with reduced viability and frequent developmental abnormalities even in recent systems (Obara et al., 2024).

Previous studies have primarily focused on optimizing culture conditions, whereas potential differences in developmental responses among chicken breeds under identical environments have not been sufficiently examined. Given that embryonic physiology and development can vary between breeds (Dao et al., 2011), such differences may also influence outcomes in eggshell-free culture.

Therefore, the objective of this Research Note was to evaluate embryo viability during culture and hatchability of White Leghorn (**WL**) and Barred Plymouth Rock (**BPR**) embryos under identical eggshell-free culture conditions and to determine whether these outcomes should be considered separately when evaluating eggshell-free culture systems.

## Materials and methods

All animal procedures were approved by the Animal Experimentation Committee, Shinshu University (Approval No. 021005). Hatched chicks were visually monitored daily after hatching in accordance with the approved animal experiment protocol. No post-hatching survival or locomotor assessment was predefined as a primary endpoint. Necropsy and veterinary examination were not performed; therefore, causes of death could not be determined.

### Experimental animals and culture conditions (pre-transplant preparation)

Fertilized eggs of WL and BPR chickens were obtained from the National Livestock Breeding Center, Okazaki Station (Okazaki, Japan). In this study, the day on which incubation was initiated was defined as incubation day 0. Source-flock and egg-handling records were obtained from the supplier. BPR eggs were obtained from the XS line and WL eggs from the MB line, and the parental flocks were approximately 72 or 74 wk of age at the time of egg collection.

Incubation was performed at 38.0°C. Eggs were automatically turned 90° every 3.75 min before transfer. Humidity was maintained by placing a water tray at the base of the incubator. Under this setup, relative humidity in our laboratory is typically maintained at approximately 60 to 80%; however, humidity was not continuously recorded with a calibrated sensor throughout the experiment.

According to supplier records, eggs were generally washed and disinfected by approximately the day after collection and stored until shipment. Fertilized eggs were stored at approximately 12°C prior to incubation, and the storage period did not exceed 1 wk.

Routine source-flock reproductive performance records and egg-quality inspection records from the same source populations were available from the supplier. However, individual egg-quality traits, such as shell thickness, albumen height, and egg composition, were not measured for each egg used in the present experiment; therefore, these variables were not included as covariates in the statistical analyses.

### Experimental animals and culture conditions (transplantation procedures and culture conditions)

Embryos were cultured using a modified eggshell-free culture system based on the method described by Tahara and Obara (2014), with minor modifications.

Egg contents were transferred at approximately 55 to 57 h of incubation (approximately HH stages 15 to 17) into culture containers consisting of rigid plastic cups covered with polymethylpentene film (Riken Technos Corp., Tokyo, Japan). Embryos were maintained in an incubator (P-008(B), Showa Furanki Co., Ltd., Saitama, Japan) at 38.0°C. After transfer, culture containers were manually rotated approximately 120° every other day while being kept on a stand tilted approximately 15 to 30°.

Under the day-0 convention used in the present manuscript, calcium supplementation was performed on incubation day 9 by adding calcium carbonate (FUJIFILM Wako Pure Chemical Corporation, Osaka, Japan) at 250 mg per 60 g of egg weight. Egg weight was measured for each egg and was used only to determine the amount of calcium carbonate supplementation; it was not included as a covariate in the statistical analyses. On incubation day 13, small openings were introduced into the covering film to facilitate gas exchange. From incubation day 16 until hatching, oxygen was supplied at a flow rate of 8 mL/min using a high-concentration oxygen generator (KO307, Kobayashi Pharmaceutical Co., Ltd., Osaka, Japan). Oxygen concentration in the culture container was additionally measured using a PreSens Fibox 4 trace under reconstructed culture conditions. Measurements were performed both in an embryo-free culture container and in a culture container containing a developing embryo on incubation day 16, corresponding to the timing of oxygen supplementation. The same type of culture container and oxygen supply condition were used as in the present study.

The embryos were cultured in two independent experiments. Both experiments included WL and BPR embryos and used the same eggshell-free culture protocol. Data from the two experiments were pooled for the primary analysis after confirming descriptively that hatching of BPR embryos was not confined to a single experiment.

#### Additional developmental observations

In addition to the primary analysis, two additional culture trials were performed to obtain descriptive developmental observations and representative embryo images under the same transfer-based eggshell-free culture conditions. These additional observations included 23 WL and 22 BPR embryos that were successfully transferred and followed at selected observation days. Representative images were obtained at transfer, incubation day 8, incubation day 10, and incubation day 12 using a Dino-Lite Edge S FLC Polarizer and DinoCapture 2.0. These observations were not included in the primary Kaplan-Meier analysis or hatchability analysis. Images were used for descriptive purposes and not for quantitative morphometric analysis.

### Definition of embryo viability during culture

Embryo viability during culture was defined based on whether embryos remained alive during the culture period. Embryo death or developmental arrest was determined at scheduled observation points by visual observation based on indicators such as absence of visible heartbeat or blood circulation, loss of embryonic movement, whitening or opacity of the embryo, vascular regression, and absence of visible developmental progression. The event day was defined as the incubation day on which embryo death or developmental arrest was first confirmed by visual observation. Culture was continued until the expected hatching time, incubation day 21. Embryos that died without hatching were stored frozen after death was confirmed. Hatching was treated as a censored observation at the day on which hatching was confirmed because hatchability was analyzed separately.

### Statistical analysis

A total of 28 embryos (13 WL and 15 BPR) were analyzed. Each embryo was considered the experimental unit. Embryo viability during culture was analyzed as time-to-event data using the Kaplan-Meier method (Kaplan and Meier, 1958). Embryo death or developmental arrest was treated as an event, whereas hatching was treated as a censored observation at the day on which hatching was confirmed. Differences in embryo viability during culture between WL and BPR embryos were evaluated using the log-rank test (Mantel, 1966). Hatchability was analyzed separately and compared between breeds using Fisher’s exact test because of the small sample size and the presence of zero hatching events in one group. Exact 95% confidence intervals for hatchability were calculated using the Clopper-Pearson method implemented in binom.test in R. Statistical significance was set at *p* < 0.05. All analyses were conducted using R (version 4.5.2; R Foundation for Statistical Computing, Vienna, Austria). Kaplan-Meier analysis and the log-rank test were performed using the survival package (version 3.8.6). Figures were generated using the survminer and ggplot2 packages.

### Hatchability

Hatchability was defined as the number of hatched embryos divided by the total number of embryos analyzed in each breed. Post-hatching survival and post-hatching locomotor ability were recorded descriptively but were not included as primary endpoints or in statistical analysis.

## Results and discussion

A total of 28 embryos were analyzed, including 13 WL embryos and 15 BPR embryos from two independent experiments. Both experiments included WL and BPR embryos. Hatching of BPR embryos was observed in both experiments (1/6 in the first experiment and 2/9 in the second experiment), whereas no WL embryos hatched in either experiment.

**Fig. 1:**
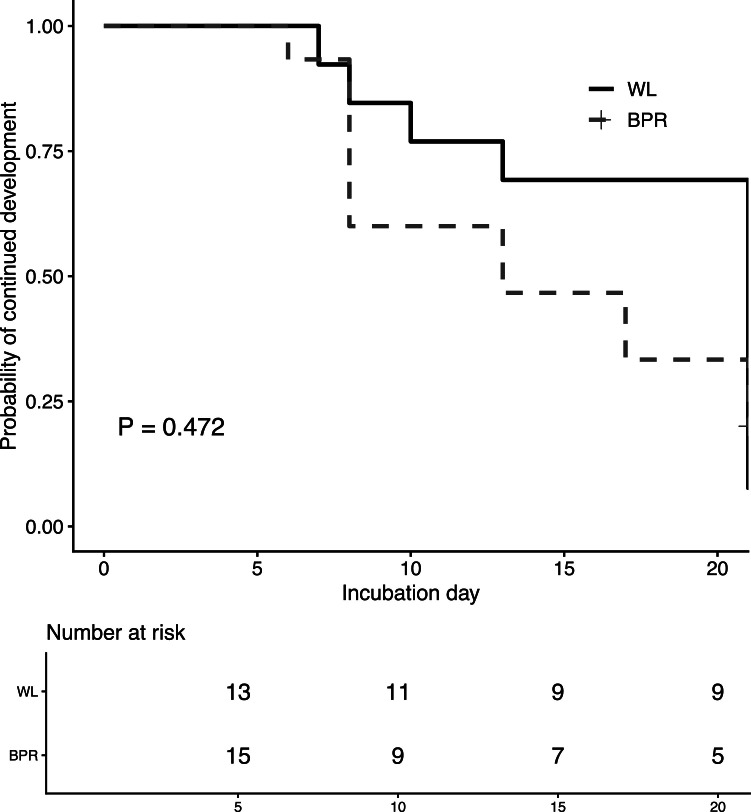
Kaplan-Meier analysis of embryo viability during culture in White Leghorn and Barred Plymouth Rock embryos under eggshell-free culture. The day on which incubation was initiated was defined as incubation day 0. Embryo death or developmental arrest was treated as an event, whereas hatching was treated as a censored observation at the day on which hatching was confirmed. The number of embryos at risk is shown below the plot. Embryo viability during culture did not differ significantly between breeds by the log-rank test (*p* = 0.472).

Embryo viability during culture was analyzed using the Kaplan-Meier method, with embryo death or developmental arrest treated as an event and hatching treated as a censored observation. Embryo viability during culture did not differ significantly between WL and BPR embryos by the log-rank test (*p* = 0.472; Fig. 1).

Hatching was observed in 3 of 15 BPR embryos (20.0%; 95% CI, 4.3-48.1%) but in none of the 13 WL embryos (0.0%; 95% CI, 0.0-24.7%). However, hatchability did not differ significantly between breeds by Fisher’s exact test (*p* = 0.226). Thus, hatching was observed only in BPR embryos, but this finding should be interpreted descriptively because of the small sample size and the lack of statistical significance.

Post-hatching observations were descriptive only. All 3 hatched BPR chicks died from undetermined causes within 2 wk after hatching. Two chicks were unable to stand or walk normally and moved by crawling, whereas one chick was able to walk and survived for approximately 2 wk. Because post-hatching survival and locomotor ability were not predefined endpoints, and because necropsy and veterinary examination were not performed, these observations were not analyzed statistically and the causes of death could not be determined.

**Fig. 2:**
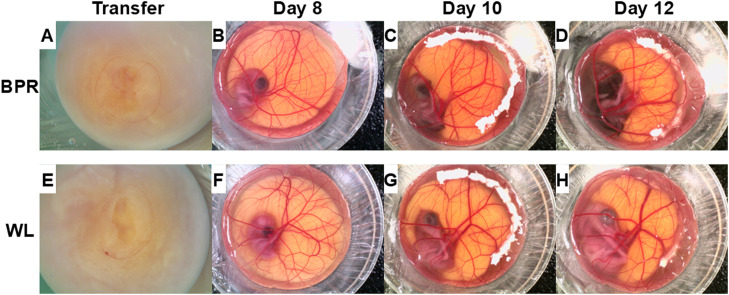
Representative images of Barred Plymouth Rock (BPR) and White Leghorn (WL) embryos during additional developmental observations under the transfer-based eggshell-free culture system. Images were obtained at transfer, incubation day 8, incubation day 10, and incubation day 12. The day on which incubation was initiated was defined as incubation day 0. Transfer images were obtained 55 to 57 h after incubation and corresponded approximately to HH stages 15 to 17. The upper row shows the same representative BPR embryo across time points (A-D), and the lower row shows the same representative WL embryo across time points (E-H). Images show gross embryonic development and extraembryonic vascularization and were used for descriptive purposes, not for quantitative morphometric analysis.

Two additional culture trials were performed to obtain descriptive developmental observations and representative embryo images under the same transfer-based eggshell-free culture conditions. These observations included 23 WL and 22 BPR embryos that were successfully transferred and followed at selected observation days. Representative images at transfer, incubation day 8, incubation day 10, and incubation day 12 are shown in Fig. 2. Live embryos available for observation in Trial 1 were 4/13 WL embryos and 6/12 BPR embryos on incubation day 8, 3/13 WL embryos and 6/12 BPR embryos on incubation day 10, and 2/13 WL embryos and 6/12 BPR embryos on incubation day 12. In Trial 2, live embryos available for observation were 2/10 WL embryos and 7/10 BPR embryos on incubation day 8, 2/10 WL embryos and 7/10 BPR embryos on incubation day 10, and 2/10 WL embryos and 6/10 BPR embryos on incubation day 12. These additional observations were not included in the primary Kaplan-Meier analysis or hatchability analysis.

In the embryo-free reconstructed setup, oxygen concentration increased from approximately 65% at the beginning of recording and reached approximately 97 to 99% after 2 to 3 h of oxygen supply, eventually stabilizing near 99 to 100%. In an additional measurement using a culture container containing a developing embryo on incubation day 16, oxygen concentration increased from approximately 86% at the beginning of recording and reached approximately 98% within 25 to 30 min. Thereafter, oxygen concentration was maintained at approximately 98 to 99%.

The present results support the separate assessment of embryo viability during culture and hatchability in eggshell-free culture studies. Although statistically significant breed differences were not detected, hatching was observed only in BPR embryos. This observation highlights the need to consider breed, genetic background, or source-flock-related factors as potential contributors in future evaluations of eggshell-free culture systems.

Several limitations should be considered. First, the sample size was limited, particularly for hatchability, and the absence of hatching in WL embryos should not be interpreted as definitive evidence of a breed difference in hatchability. Second, only two breeds and specific source lines were examined; therefore, the findings should not be generalized to chicken breeds broadly. Third, although additional source-flock and egg-handling records were obtained from the supplier, individual egg-quality traits, such as shell thickness, albumen height, and egg composition, were not measured for each egg used in the present experiment. These variables, together with parental flock age, storage-related factors, and other source-flock factors, could not be included as covariates because of the limited sample size. Fourth, humidity was not continuously recorded with a calibrated sensor throughout the experiment. Oxygen concentrations were additionally measured under reconstructed culture conditions, including an embryo-free container and a container containing a developing embryo on incubation day 16; however, these were not direct continuous measurements during the original embryo culture experiment and should be interpreted as approximate oxygen concentrations achieved under the culture setup rather than precise measurements of the local oxygen environment around each embryo. Fifth, the results were obtained under a specific eggshell-free culture protocol based on Tahara and Obara (2014), with minor modifications in the timing of culture manipulations, and the individual effects of culture interventions such as calcium carbonate supplementation, film openings for gas exchange, and oxygen supply were not evaluated separately. In addition, post-hatching survival and long-term chick viability were not evaluated as primary endpoints in the present study. All 3 hatched chicks died from undetermined causes within 2 wk after hatching, and 2 of the 3 showed impaired locomotion; therefore, hatchability in this study should be interpreted as successful hatching from eggshell-free culture rather than evidence of sustained post-hatching viability. Future studies comparing eggshell-free culture with other avian embryo culture systems, including direct *ex ovo* culture from the beginning of incubation, conventional in-shell incubation, and surrogate eggshell culture, will be necessary to determine whether strain- or source-related differences in embryo viability and hatchability depend on the culture system.

In conclusion, embryo viability during culture and hatchability did not differ significantly between WL and BPR embryos under the present eggshell-free culture conditions. However, hatching was observed only in BPR embryos, whereas several WL embryos remained viable until late incubation without hatching. These results support the separate assessment of embryo viability during culture and hatchability and highlight the need to consider breed, genetic background, or source-flock-related factors as potential contributors in future eggshell-free culture studies. Because all hatched chicks died within 2 wk after hatching, hatchability in the present study should not be interpreted as evidence of sustained post-hatching viability or practical post-hatching utility.

## Declaration of AI and AI-assisted Technologies in the Writing Process

During the preparation of this work, the authors used DeepL for language editing and translation support, Consensus for literature-search support, and ChatGPT for language editing, structural refinement, and consistency checking between the manuscript text and statistical outputs. After using these tools, the authors reviewed and edited the content as needed and take full responsibility for the content of the publication. No AI-assisted tool was used to generate or alter the data or to perform the statistical analyses. All statistical analyses were conducted by the authors using R, and the authors verified all data, results, and interpretations.

## CRediT authorship contribution statement

**Kie Murai:** Writing – original draft, Visualization, Validation, Software, Resources, Methodology, Investigation, Formal analysis, Data curation, Conceptualization. **Hiroshi Kagami:** Writing – review & editing, Supervision, Resources, Project administration, Funding acquisition.

## Disclosures

The authors declare no conflict of interest.
